# Experiences of African American Dementia Caregivers During the COVID-19 Pandemic

**DOI:** 10.1093/geroni/igab046.3482

**Published:** 2021-12-17

**Authors:** Sloan Oliver, Karah Alexander, Fayron Epps

**Affiliations:** Emory University, Atlanta, Georgia, United States

## Abstract

African American caregivers are often confronted with the complexities of caregiving through the lens of race and associated health disparities. The COVID-19 pandemic has both exacerbated the systemic disparities and deeply rooted inequities experienced by African Americans and laid bare their effects on the community of caregivers. The purpose of this project was to explore the experiences of African American dementia caregivers during the COVID-19 pandemic. Nineteen African American caregivers of persons living with dementia were recruited by primary investigators and community partners with purposeful sampling techniques to participate in semi-structured focus groups that were held April 2021. Four overarching themes were constructed during thematic analysis: social isolation, decreased well-being, the good and bad of telehealth, and challenges in fulfilling the caregiver role. Caregivers expressed that they became socially isolated from family and friends, which led to them becoming depressed and mentally strained. Several caregivers felt they could not carry out their caregiver duties due to the constraints surrounding the pandemic. The varying levels of interaction with and the comfort level of physicians utilizing telehealth led to caregivers having mixed reviews on the popularized service. The results of this study will be used to culturally adapt caregiving education courses and programs promoting mastery and competency during a pandemic. In preparations for future public health crises, healthcare professionals will be able to use the results of this study to address the specific needs and improve the experiences of African American dementia caregivers.

